# Study on the interior equilibrium point of a special class of 2 × 2 × 2 asymmetric evolutionary games

**DOI:** 10.1098/rsos.231960

**Published:** 2024-07-24

**Authors:** Sha Song, Qiuhui Pan, Mingfeng He

**Affiliations:** ^1^ School of Mathematical Sciences, Dalian University of Technology, Dalian 116024, People’s Republic of China; ^2^ School of Innovation and Entrepreneurship, Dalian University of Technology, Dalian 116024, People’s Republic of China

**Keywords:** evolutionary games, 2 × 2 × 2 asymmetric games, interior equilibrium point, stability, generalized Hamiltonian system

## Abstract

Many behavioural interactions in real life involve three individuals. When each individual has two alternative strategies, they can be abstracted into mathematical models by means of 2×2×2 asymmetric games. In this paper, we explore a special class of 2×2×2 asymmetric games satisfying fixed conditions. Firstly, we analyse two solitary interior equilibrium points and provide the judgement condition for their instability based on the Jacobi matrix local stability analysis method. Secondly, we analyse the interior equilibrium points that are continuously distributed within a line and probe into their stability conditions based on generalized Hamiltonian systems theory. Under the circumstances, the stable interior equilibrium point is surrounded by closed orbits in phase space, which presents an observable stable state where two strategies coexist and fluctuate in each of the three game populations. This work enriches the study of 2×2×2 asymmetric games’ evolutionary dynamics.

## 1. Introduction

Evolutionary games can be simply classified as symmetric games [1] and asymmetric games [2]. Compared with the former, the latter breaks the assumption of homogeneous game populations and is more general. Asymmetric games can be used to describe not only the game between different populations but also the game between different types of individuals within the same population [3–6]. The fact that there are no two identical individuals and different roles in the same event result in individuals being assigned different values indicates that asymmetric games are normative. Formally, symmetric games can be regarded as a special type of asymmetric games, where the non-strategic characteristics of players are consistent and the payoff matrix of players is the same. Asymmetric games may also be simplified into specific symmetric games in certain situations [7,8]. Tuyls *et al*. [9] decomposed asymmetric games into two single-population symmetric games and proved that the Nash equilibrium of asymmetric games corresponds to the combination of the Nash equilibrium of two symmetric games. Owing to the simpler mathematical form and ease of analysis, symmetric games are favoured by game theory researchers. However, compared with symmetric games, asymmetric games are more relevant to experimental research and can describe more general real-life examples to solve a wider range of application problems, such as volunteer’s dilemma game [10–13] and public goods games [14] with asymmetric costs and unequal payoffs. In recent years, some scholars have expanded the theoretical research in symmetric games to asymmetric games. For example, stochastic evolutionary dynamics of asymmetric games in finite populations [15–20] and zero-determinant strategies in asymmetric games [21,22].

According to the number of participants in a game, asymmetric games can be divided into two-party games and multi-party games. When each player has two alternative strategies, they can be further expressed as 2×2 asymmetric games (two parties), 2×2×2 asymmetric games (three parties) and so on. In the early stages of asymmetric games, theoretical research on two-party asymmetric games has been paid attention to, especially the mixed strategy (interior equilibrium point). Through static analysis, Selten [23] proved that there is no mixed evolutionary stable strategy in two-party asymmetric games. Through dynamic analysis, Hofbauer [24] indicated that there cannot be any asymptotically stable fixed points or other attractors in the state space of two-party asymmetric games. These show that in the two-party asymmetric evolutionary games, only pure strategy can converge to the original positions after interference (in fact, under certain parameter values, the mixed strategy (interior equilibrium point) is the centre point surrounded by closed orbits [24–26]). To this end, Binmore & Samuelson [27] enriched Selten’s model, assuming that both payoffs and asymmetry may be imperfectly observed, and found the stability conditions of effective mixed strategies (interior equilibrium points) in asymmetric games. Sáez-Marti [28] combined the best response dynamics to modify the standard replicator dynamics and proved through the Lyapunov function that, under the behavioural evolution mechanism of this joint dynamics, the interior equilibrium points of 2×2 asymmetric games can be asymptotically converged. In addition to the study of interior equilibrium points, Song *et al*. [29] conducted stability discussions on boundary equilibrium points of 2×2 asymmetric games. Some achievements have been made in the study of two-party asymmetric games. Besides, higher order interactions have been noticed as well [30–33], but most of these studies focus on multi-party symmetric games. Here, this paper will probe into the simplest form of multi-party asymmetric games, namely 2×2×2 asymmetric games involving three parties. The dynamic properties of face equilibrium points and edge equilibrium points in 2×2×2 asymmetric games have been explored [34], and we will continue to discuss those of interior equilibrium points. Actually, as a mathematical analysis tool, 2×2×2 asymmetric games have been widely used in practical problems, such as carbon emission reduction mechanisms, including carbon emission trading [35–37] and carbon-sink fishery [38,39], environmental mass incidents [40], supply chains [41,42], medical consortiums [43] and so on. In the process of analysing the model, these works simply rely on Jacobi matrix local stability analysis method to discuss the stability of pure strategies in 2×2×2 asymmetric games system, which may ignore other important conclusions.

We explore the dynamic properties of 2×2×2 asymmetric evolutionary games by means of generalized Hamiltonian system theory. The classical Hamiltonian system is a conservative system, and the corresponding theoretical knowledge has been used for 2×2 asymmetric games [24,26], but it can only be applied to even dimensional systems. With the development of scientific research and the deepening understanding of nonlinear dynamical systems, the behaviour evolution of a large number of odd-dimensional systems needs to be studied. Furthermore, odd-dimensional Hamiltonian systems are called generalized Hamiltonian systems, and the Hamiltonian structure of three-dimensional dynamical systems is also known as bi-Hamiltonian structures. In the light of generalized Hamiltonian system theory, different types of three-dimensional dynamical systems have been analysed, such as L$\overset{¨}{u}$ systems, Chen systems and Qi systems known for their chaotic characteristics [44], optical Maxwell–Bloch equations [45], epidemiological Kermack–McKendrick models [46], Lotka–Volterra equations describing species interactions in ecosystems [47,48] and tournaments based on replicator equations [49]. In recent years, research on the Hamiltonian characteristics of three-dimensional dynamical systems has also been going deep [50–55]. Based on generalized Hamiltonian system theory, we probe into the dynamic properties of a special class of 2×2×2 asymmetric evolutionary games that meet certain conditions, that is, to discuss the existence and stability of interior equilibrium points, and the stability here refers to Lyapunov stability. Finally, an example is given to more intuitively reflect the conclusions obtained in this paper.

## 2. Model

In 2×2×2 asymmetric games, suppose there are three populations, i.e. population A, population B and population C. Each population has two strategies, that is, the strategy set of population A is $H_{A}=\{A_{1},A_{2}\}$, the strategy set of population B is $H_{B}=\{B_{1},B_{2}\}$ and the strategy set of population C is $H_{C}=\{C_{1},C_{2}\}$. The payoff matrix is shown in table 1.

**Table 1.** Payoff matrix of 2×2×2 asymmetric games.

		B	C	
			$C_{1}$	$C_{2}$
A	$A_{1}$	$B_{1}$	$\left(a_{1},b_{1},c_{1}\right)$	$\left(a_{2},b_{2},c_{2}\right)$
		$B_{2}$	$\left(a_{3},b_{3},c_{3}\right)$	$\left(a_{4},b_{4},c_{4}\right)$
	$A_{2}$	$B_{1}$	$\left(a_{5},b_{5},c_{5}\right)$	$\left(a_{6},b_{6},c_{6}\right)$
		$B_{2}$	$\left(a_{7},b_{7},c_{7}\right)$	$\left(a_{8},b_{8},c_{8}\right)$

In fact, the payoff matrix described in table 1 can be equitably represented by the following matrices [34]:


(2.1) $$M_{A}=\left(\begin{array}{cc} a_{11} & a_{12}\\ a_{21} & a_{22} \end{array}\right),M_{B}=\left(\begin{array}{cc} b_{11} & b_{12}\\ b_{21} & b_{22} \end{array}\right),M_{C}=\left(\begin{array}{cc} c_{11} & c_{12}\\ c_{21} & c_{22} \end{array}\right),$$

where $a_{11}=a_{1}-a_{5}$, $a_{12}=a_{2}-a_{6}$, $a_{21}=a_{3}-a_{7}$, $a_{22}=a_{4}-a_{8}$; $b_{11}=b_{1}-b_{3}$, $b_{12}=b_{5}-b_{7}$, $b_{21}=b_{2}-b_{4}$, $b_{22}=b_{6}-b_{8}$; $c_{11}=c_{1}-c_{2}$, $c_{12}=c_{3}-c_{4}$, $c_{21}=c_{5}-c_{6}$, $c_{22}=c_{7}-c_{8}$. $M_{A}$, $M_{B}$ and $M_{C}$ are payoff difference matrices of population A, population B and population C, respectively.

Suppose that the proportion of strategy $A_{1}$ in the population A is x, then the proportion of strategy $A_{2}$ is 1−x. Suppose that the proportion of strategy $B_{1}$ in the population B is y, then the proportion of strategy $B_{2}$ is 1−y. Suppose that the proportion of strategy $C_{1}$ in the population C is z, then the proportion of strategy $C_{2}$ is 1−z. Combining payoff difference matrices given in equation (2.1), we can obtain replicator equations of 2×2×2 asymmetric games,


(2.2) $$\{\begin{array}{c} \dot{x}=x(1-x)f(y,z)\\ \dot{y}=y(1-y)g(z,x)\\ \dot{z}=z(1-z)h(x,y) \end{array},$$

where


$$\begin{array}{rl} f(y,z) & =a_{11}yz+a_{12}y(1-z)+a_{21}(1-y)z+a_{22}(1-y)(1-z)\\ & =(a_{11}-a_{12}-a_{21}+a_{22})yz+(a_{12}-a_{22})y+(a_{21}-a_{22})z+a_{22},\\ g(z,x) & =b_{11}zx+b_{12}z(1-x)+b_{21}(1-z)x+b_{22}(1-z)(1-x)\\ & =(b_{11}-b_{12}-b_{21}+b_{22})zx+(b_{12}-b_{22})z+(b_{21}-b_{22})x+b_{22},\\ h(x,y) & =c_{11}xy+c_{12}x(1-y)+c_{21}(1-x)y+c_{22}(1-x)(1-y)\\ & =(c_{11}-c_{12}-c_{21}+c_{22})xy+(c_{12}-c_{22})x+(c_{21}-c_{22})y+c_{22}. \end{array}$$

Generalizing from Hofbauer’s bi-matrix work [25], for 2×2×2 asymmetric games, there are six equations with three redundant equations. See appendix A and Song *et al*. [34] for the detailed derivation process of equation (2.2).

Let $\dot{x}=0$, $\dot{y}=0$, $\dot{z}=0$, it follows that the equilibrium point of 2×2×2 asymmetric games evolution system is $E=(x^{*},y^{*},z^{*})\in {\left[0,1\right]}^{3}$. Furthermore, we name E interior equilibrium point, if $E=(x^{*},y^{*},z^{*})\in {\left(0,1\right)}^{3}$. Substituting the interior equilibrium point into functions f(y,z), g(z,x), h(x,y), obviously, one can obtain that f=0, g=0, h=0. In other words, if an interior equilibrium point exists, it must be a solution of f=0, g=0 and h=0. The following will mainly discuss functions f(y,z), g(z,x) and h(x,y). For the convenience of solving the interior equilibrium point, we provide a proposition.

***Proposition 1****. When the payoff difference matrix* $M_{A}$ *satisfies the condition that* $|M_{A}|=0$*,* f(y,z) *can be formalized as* $k_{f}\cdot (A_{f}y+B_{f})\cdot (C_{f}z+D_{f})$*, i.e.* $f(y,z)=\frac{1}{a_{22}}((a_{12}-a_{22})y+a_{22})((a_{21}-a_{22})z+a_{22})$. *When the payoff difference matrix* $M_{B}$ *satisfies the condition that* $|M_{B}|=0$*,* g(z,x) *can be formalized as* $k_{g}\cdot (A_{g}z+B_{g})\cdot (C_{g}x+D_{g})$, *i.e.* $g(z,x)=\frac{1}{b_{22}}((b_{12}-b_{22})z+b_{22})((b_{21}-b_{22})x+b_{22})$. *When the payoff difference matrix* $M_{C}$ *satisfies the condition that* $|M_{C}|=0$*,* h(x,y) *can be formalized as* $k_{h}\cdot (A_{h}x+B_{h})\cdot (C_{h}y+D_{h})$, *i.e.* $h(x,y)=\frac{1}{c_{22}}((c_{12}-c_{22})x+c_{22})((c_{21}-c_{22})y+c_{22})$.

*Proof*. It follows that $a_{11}a_{22}=a_{12}a_{21}$ from $|M_{A}|=0$; thus, we have


$$\begin{array}{ll} f\left(y,z\right) & =\frac{1}{a_{22}}\left(\left(a_{12}-a_{22}\right)y+a_{22}\right)\left(\left(a_{21}-a_{22}\right)z+a_{22}\right)\\ & =\frac{1}{a_{22}}\left(\left(a_{12}a_{21}-a_{12}a_{22}-a_{21}a_{22}+a_{22}^{2}\right)yz+\left(a_{12}-a_{22}\right)a_{22}y+\left(a_{21}-a_{22}\right)a_{22}z+a_{22}^{2}\right)\\ & =\left(a_{11}-a_{12}-a_{21}+a_{22}\right)yz+\left(a_{12}-a_{22}\right)y+\left(a_{21}-a_{22}\right)z+a_{22}. \end{array}$$

The proof process for the remaining two is similar.

∎

From proposition 1, when each determinant of three payoff difference matrices given in equation (2.1) is equal to zero, that is, when $|M_{A}|=0$, $|M_{B}|=0$, $|M_{C}|=0$, equation (2.2) becomes


(2.3) $$\{\begin{array}{c} \dot{x}=x(1-x)f(y,z)\\ \dot{y}=y(1-y)g(z,x)\\ \dot{z}=z(1-z)h(x,y) \end{array},$$

where


$$\begin{array}{c} f(y,z)=\frac{1}{a_{22}}((a_{12}-a_{22})y+a_{22})((a_{21}-a_{22})z+a_{22}),\\ g(z,x)=\frac{1}{b_{22}}((b_{12}-b_{22})z+b_{22})((b_{21}-b_{22})x+b_{22}),\\ h(x,y)=\frac{1}{c_{22}}((c_{12}-c_{22})x+c_{22})((c_{21}-c_{22})y+c_{22}). \end{array}$$

Actually, if equation (2.3) has an interior equilibrium point, it is easy to find. Next, this paper will pay attention to equation (2.3), that is, a special class of 2×2×2 asymmetric evolutionary games that satisfy certain fixed conditions. We analyse the existence and stability of interior equilibrium points of equation (2.3), with the help of Jacobi matrix local stability analysis method and generalized Hamiltonian system theory.

### 2.1. Interior equilibrium points

Let f=0, g=0 and h=0 in equation (2.3). We have equilibrium points


$$\left(x^{*},y^{*},z^{*})=(\frac{c_{22}}{c_{22}-c_{12}},\frac{a_{22}}{a_{22}-a_{12}},\frac{b_{22}}{b_{22}-b_{12}}\right)$$

and


$$(x^{**},y^{**},z^{**})=(\frac{b_{22}}{b_{22}-b_{21}},\frac{c_{22}}{c_{22}-c_{21}},\frac{a_{22}}{a_{22}-a_{21}}).$$

In the context of 2×2×2 asymmetric games, these two points exist and are interior equilibrium points of equation (2.3) only when each component value falls within the range (0,1), which is demonstrated through the following proposition.

***Proposition 2****. When the system given in* *equation (2.3)* *satisfies the condition*


(2.4) $$c_{12}c_{22}<0,\;a_{12}a_{22}<0,\;b_{12}b_{22}<0,$$

*the equilibrium point*


$$\left(x^{*},y^{*},z^{*})=(\frac{c_{22}}{c_{22}-c_{12}},\frac{a_{22}}{a_{22}-a_{12}},\frac{b_{22}}{b_{22}-b_{12}}\right)$$

*is an interior equilibrium point.*

*Proof*. When each component value falls within the range (0,1), the equilibrium point


$$\left(x^{*},y^{*},z^{*})=(\frac{c_{22}}{c_{22}-c_{12}},\frac{a_{22}}{a_{22}-a_{12}},\frac{b_{22}}{b_{22}-b_{12}}\right)$$

is an interior equilibrium point, and vice versa.

Owing to


$$\begin{array}{c} c_{12}c_{22}<0⟺0<\frac{c_{22}}{c_{22}-c_{12}}<1,\\ a_{12}a_{22}<0⟺0<\frac{a_{22}}{a_{22}-a_{12}}<1,\\ b_{12}b_{22}<0⟺0<\frac{b_{22}}{b_{22}-b_{12}}<1, \end{array}$$

it can be drawn that when the system given in equation (2.3) satisfies the condition given in equation (2.4), the equilibrium point $\left(x^{*},y^{*},z^{*}\right)$ is an interior equilibrium point.

∎

Similarly, when the system given in equation (2.3) satisfies the condition,


$$b_{21}b_{22}<0,\;c_{21}c_{22}<0,\;a_{21}a_{22}<0,$$

the equilibrium point


$$\left(x^{**},y^{**},z^{**})=(\frac{b_{22}}{b_{22}-b_{21}},\frac{c_{22}}{c_{22}-c_{21}},\frac{a_{22}}{a_{22}-a_{21}}\right)$$

is an interior equilibrium point.

What is more, the stability of the interior equilibrium point $\left(\frac{c_{22}}{c_{22}-c_{12}},\frac{a_{22}}{a_{22}-a_{12}},\frac{b_{22}}{b_{22}-b_{12}}\right)$ is discussed as follows.

***Theorem 1*.** *When the system given in* *equation (2.3)* *satisfies the condition given in* *equation (2.4)**, and if the inequalities*


(2.5) $$a_{12}c_{22}\neq a_{22}c_{21},b_{12}a_{22}\neq b_{22}a_{21},c_{12}b_{22}\neq c_{22}b_{21}$$

*are satisfied, then the interior equilibrium point*


$$\left(x^{*},y^{*},z^{*})=(\frac{c_{22}}{c_{22}-c_{12}},\frac{a_{22}}{a_{22}-a_{12}},\frac{b_{22}}{b_{22}-b_{12}}\right)$$

*is unstable.*

*Proof*. Let


$$F(x,y,z)=\dot{x},\;G(x,y,z)=\dot{y},\;H(x,y,z)=\dot{z},$$

we have the Jacobi matrix about F(x,y,z), G(x,y,z) and H(x,y,z), that is,


(2.6) $$Jacobi=\left(\begin{array}{ccc} \frac{\partial F}{\partial x} & \frac{\partial F}{\partial y} & \frac{\partial F}{\partial z}\\ \frac{\partial G}{\partial x} & \frac{\partial G}{\partial y} & \frac{\partial G}{\partial z}\\ \frac{\partial H}{\partial x} & \frac{\partial H}{\partial y} & \frac{\partial H}{\partial z} \end{array}\right),$$

where


$$\frac{\partial F}{\partial x}=-2(x-\frac{1}{2})f(y,z),\;\;\;\frac{\partial F}{\partial y}=x(1-x)\frac{\left(a_{12}-a_{22})((a_{21}-a_{22})z+a_{22}\right)}{a_{22}},$$

$$\frac{\partial F}{\partial z}=x(1-x)\frac{\left(a_{21}-a_{22})((a_{12}-a_{22})y+a_{22}\right)}{a_{22}},\;\;\;\frac{\partial G}{\partial x}=y(1-y)\frac{\left(b_{21}-b_{22})((b_{12}-b_{22})z+b_{22}\right)}{b_{22}},$$

$$\frac{\partial G}{\partial y}=-2(y-\frac{1}{2})g(z,x),\;\;\;\frac{\partial G}{\partial z}=y(1-y)\frac{\left(b_{12}-b_{22})((b_{21}-b_{22})x+b_{22}\right)}{b_{22}},$$

$$\frac{\partial H}{\partial x}=z(1-z)\frac{\left(c_{12}-c_{22})((c_{21}-c_{22})y+c_{22}\right)}{c_{22}},\;\;\;\frac{\partial H}{\partial y}=z(1-z)\frac{\left(c_{21}-c_{22})((c_{12}-c_{22})x+c_{22}\right)}{c_{22}},$$

$$\frac{\partial H}{\partial z}=-2(z-\frac{1}{2})h(x,y).$$

The Jacobi matrix at the interior equilibrium point


$$E_{in}=\left(x^{*},y^{*},z^{*}\right)=\left(\frac{c_{22}}{c_{22}-c_{12}},\frac{a_{22}}{a_{22}-a_{12}},\frac{b_{22}}{b_{22}-b_{12}}\right)$$

is


(2.7) $$Jacobi{|}_{E_{in}}=\left(\begin{array}{ccc} 0 & -\frac{c_{22}c_{12}(a_{12}-a_{22})(a_{22}b_{12}-b_{22}a_{21})}{a_{22}(c_{22}-c_{12}{)}^{2}(b_{12}-b_{22})} & 0\\ 0 & 0 & -\frac{a_{22}a_{12}(b_{12}-b_{22})(b_{22}c_{12}-c_{22}b_{21})}{b_{22}(a_{22}-a_{12}{)}^{2}(c_{12}-c_{22})}\\ -\frac{b_{22}b_{12}(c_{12}-c_{22})(c_{22}a_{12}-a_{22}c_{21})}{c_{22}(b_{22}-b_{12}{)}^{2}(a_{12}-a_{22})} & 0 & 0 \end{array}\right),$$

thus, we have eigenvalues


(2.8) $$\lambda_{1}=\delta ,\lambda_{2}=\frac{-1-\sqrt{3}i}{2}\delta ,\lambda_{3}=\frac{-1+\sqrt{3}i}{2}\delta ,$$

where$\delta =\frac{\sqrt[{3}]{-a_{12}b_{12}c_{12}(a_{12}-a_{22}{)}^{4}(b_{12}-b_{22}{)}^{4}(c_{12}-c_{22}{)}^{4}(a_{12}c_{22}-a_{22}c_{21})(b_{12}a_{22}-b_{22}a_{21})(c_{12}b_{22}-c_{22}b_{21})}}{(a_{12}-a_{22}{)}^{2}(b_{12}-b_{22}{)}^{2}(c_{12}-c_{22}{)}^{2}},i=\sqrt{-1}.$

From proposition 2, it can be known that $a_{12}\neq 0$, $b_{12}\neq 0$, $c_{12}\neq 0$, $a_{12}-a_{22}\neq 0$, $b_{12}-b_{22}\neq 0$, $c_{12}-c_{22}\neq 0$. Combining with equation (2.5), that is, $a_{12}c_{22}-a_{22}c_{21}\neq 0$, $b_{12}a_{22}-b_{22}a_{21}\neq 0$ and $c_{12}b_{22}-c_{22}b_{21}\neq 0$, it turns out that δ≠0. Furthermore, the real part of the eigenvalue $\lambda_{1}$ and the real part of the eigenvalue $\lambda_{2}$ (or the eigenvalue $\lambda_{3}$) must be different signs. Thus, the interior equilibrium point $\left(\frac{c_{22}}{c_{22}-c_{12}},\frac{a_{22}}{a_{22}-a_{12}},\frac{b_{22}}{b_{22}-b_{12}}\right)$ is unstable.

∎

Additionally, if the inequalities given in equation (2.5) are satisfied, then the interior equilibrium point


$$\left(x^{**},y^{**},z^{**})=(\frac{b_{22}}{b_{22}-b_{21}},\frac{c_{22}}{c_{22}-c_{21}},\frac{a_{22}}{a_{22}-a_{21}}\right)$$

is unstable as well.

Next, we consider the dynamic properties of the system given in equation (2.3) when equation (2.5) is not established, that is, when at least one of the equalities,


$$\begin{array}{c} a_{12}c_{22}=a_{22}c_{21},\\ b_{12}a_{22}=b_{22}a_{21},\\ c_{12}b_{22}=c_{22}b_{21}, \end{array}$$

is established.

In fact, when equation (2.5) does not hold, all three eigenvalues in equation (2.8) are zero, and the stability of the interior equilibrium point $\left(\frac{c_{22}}{c_{22}-c_{12}},\frac{a_{22}}{a_{22}-a_{12}},\frac{b_{22}}{b_{22}-b_{12}}\right)$ cannot be determined here. Similarly, the stability of the interior equilibrium point $(\frac{b_{22}}{b_{22}-b_{21}},\frac{c_{22}}{c_{22}-c_{21}},$$\frac{a_{22}}{a_{22}-a_{21}})$ cannot be determined as well. But interestingly, when equation (2.5) does not hold, there may be an infinite number of interior equilibrium points in the system given in equation (2.3), i.e. one of the component values of interior equilibrium points $\left(\frac{c_{22}}{c_{22}-c_{12}},\frac{a_{22}}{a_{22}-a_{12}},\frac{b_{22}}{b_{22}-b_{12}}\right)$ or $\left(\frac{b_{22}}{b_{22}-b_{21}},\frac{c_{22}}{c_{22}-c_{21}},\frac{a_{22}}{a_{22}-a_{21}}\right)$ is considered as any number within the range (0,1), such as points $\left(\tilde{x},\frac{a_{22}}{a_{22}-a_{12}},\frac{b_{22}}{b_{22}-b_{12}}\right)$ with $\tilde{x}\in (0,1)$ and points $\left(\frac{c_{22}}{c_{22}-c_{12}},\tilde{y},\frac{b_{22}}{b_{22}-b_{12}}\right)$ with $\tilde{y}\in (0,1)$. The discussion in this paper takes the points $\left(\tilde{x},\frac{a_{22}}{a_{22}-a_{12}},\frac{b_{22}}{b_{22}-b_{12}}\right)$ with $\tilde{x}\in (0,1)$ as an example, and other similar points have the same analysis process. The following is given in the form of a theorem to judge whether points $\left(\tilde{x},\frac{a_{22}}{a_{22}-a_{12}},\frac{b_{22}}{b_{22}-b_{12}}\right)$ with $\tilde{x}\in (0,1)$ are interior equilibrium points.

***Theorem 2******.** When* *equation (2.3)* *satisfies the conditions,*


(2.9) $$a_{12}c_{22}=a_{22}c_{21},$$

*and*


(2.10) $$a_{12}a_{22}<0,b_{12}b_{22}<0,$$

*for* $\forall \tilde{x}\in (0,1)$, *the point* $\left(\tilde{x},\frac{a_{22}}{a_{22}-a_{12}},\frac{b_{22}}{b_{22}-b_{12}}\right)$ *is an interior equilibrium point.*

*Proof*. It follows that f(y,z)=0 from $y=\frac{a_{22}}{a_{22}-a_{12}}$, and it follows that g(z,x)=0 from $z=\frac{b_{22}}{b_{22}-b_{12}}$. Owing to


$$a_{12}c_{22}=a_{22}c_{21},$$

we have,


$$\frac{\left(a_{12}-a_{22}\right)y\;+\;a_{22}}{\left(c_{21}-c_{22}\right)y\;+\;c_{22}}=\frac{a_{12}y\;+\;a_{22}\left(1-y\right)}{c_{21}y\;+\;c_{22}\left(1-y\right)}=\frac{\frac{a_{22}c_{21}}{c_{22}}y\;+\;a_{22}\left(1-y\right)}{c_{21}y\;+\;c_{22}\left(1-y\right)}=\frac{a_{22}}{c_{22}}\cdot \frac{c_{21}y+c_{22}\left(1-y\right)}{c_{21}y\;+\;c_{22}\left(1-y\right)}=\frac{a_{22}}{c_{22}},$$

further,


$$\frac{1}{a_{22}}\left(\left(a_{12}-a_{22}\right)y+a_{22}\right)=\frac{1}{c_{22}}\left(\left(c_{21}-c_{22}\right)y+c_{22}\right).$$

Thus, h(x,y) can be written equivalently as


$$h(x,y)=\frac{1}{a_{22}}((c_{12}-c_{22})x+c_{22})((a_{12}-a_{22})y+a_{22}).$$

According to $y=\frac{a_{22}}{a_{22}-a_{12}}$, we have h(x,y)=0.

To sum up, for $\forall \tilde{x}\in (0,1)$, the point $\left(\tilde{x},\frac{a_{22}}{a_{22}-a_{12}},\frac{b_{22}}{b_{22}-b_{12}}\right)$ is an equilibrium point.

Furthermore, owing to


$$a_{12}a_{22}<0⟺0<\frac{a_{22}}{a_{22}-a_{12}}<1$$

and


$$b_{12}b_{22}<0⟺0<\frac{b_{22}}{b_{22}-b_{12}}<1,$$

it follows that for $\forall \tilde{x}\in (0,1)$, the point $\left(\tilde{x},\frac{a_{22}}{a_{22}-a_{12}},\frac{b_{22}}{b_{22}-b_{12}}\right)$ is an interior equilibrium point.

∎

In effect, when equation (2.3) satisfies the condition given in equation (2.9), if conditions that $b_{21}b_{22}<0$ and $c_{21}c_{22}<0$ are satisfied simultaneously, then for $\forall \tilde{z}\in (0,1)$, the point $\left(\frac{b_{22}}{b_{22}-b_{21}},\frac{c_{22}}{c_{22}-c_{21}},\tilde{z}\right)$ is also an interior equilibrium point. In addition, the condition for determining the existence of other interior equilibrium points can be obtained. For instance, when equation (2.3) satisfies the condition that $c_{12}b_{22}=c_{22}b_{21}$, if conditions that $c_{12}c_{22}<0$ and $a_{12}a_{22}<0$ are satisfied simultaneously, then for $\forall \tilde{z}\in (0,1)$, the point $\left(\frac{c_{22}}{c_{22}-c_{12}},\frac{a_{22}}{a_{22}-a_{12}},\tilde{z}\right)$ is an interior equilibrium point; if conditions that $a_{21}a_{22}<0$ and $b_{21}b_{22}<0$ are satisfied simultaneously, then for $\forall \tilde{y}\in (0,1)$, the point $\left(\frac{b_{22}}{b_{22}-b_{21}},\tilde{y},\frac{a_{22}}{a_{22}-a_{21}}\right)$ is an interior equilibrium point. When equation (2.3) satisfies the condition that $b_{12}a_{22}=b_{22}a_{21}$, if conditions that $b_{12}b_{22}<0$ and $c_{12}c_{22}<0$ are satisfied simultaneously, then for $\forall \tilde{y}\in (0,1)$, the point $\left(\frac{c_{22}}{c_{22}-c_{12}},\tilde{y},\frac{b_{22}}{b_{22}-b_{12}}\right)$ is an interior equilibrium point; if conditions that $c_{21}c_{22}<0$ and $a_{21}a_{22}<0$ are satisfied simultaneously, then for $\forall \tilde{x}\in (0,1)$, the point $\left(\tilde{x},\frac{c_{22}}{c_{22}-c_{21}},\frac{a_{22}}{a_{22}-a_{21}}\right)$ is an interior equilibrium point.

### 2.2. Generalized Hamiltonian system

In this paper, generalized Hamiltonian system theory is applied to analyse the behavioural evolution dynamics in the 2×2×2 asymmetric games system. In this section, we first introduce some concepts about the generalized Hamiltonian system, including generalized Poisson brackets, skew-symmetric Poisson matrix, Poisson vector and Hamiltonian function, mainly referred to in Esen *et al*. [44]. Here we present a general three-dimensional nonlinear system


(2.11) $$\overset{˙}{u}=F\left(u\right),u=\left(x,y,z\right).$$

Generalized Poisson bracket is a binary operation {⋅,⋅} on the space of real-valued smooth functions satisfying the Leibnitz rule and the Jacobi identities. Generalized Poisson brackets are often represented as


(2.12) {F,H}=∇F⋅N∇H,

where F and H are two real-valued functions and ∇F and ∇H are gradients of F and H, respectively. N is a skew-symmetric matrix, that is,


(2.13) $$N=\left(\begin{array}{ccc} 0 & -j_{3} & j_{2}\\ j_{3} & 0 & -j_{1}\\ -j_{2} & j_{1} & 0 \end{array}\right),$$

and satisfies Jacobi identities


(2.14) $$j_{2}\frac{\partial j_{3}}{\partial x}-j_{3}\frac{\partial j_{2}}{\partial x}+j_{3}\frac{\partial j_{1}}{\partial y}-j_{1}\frac{\partial j_{3}}{\partial y}+j_{1}\frac{\partial j_{2}}{\partial z}-j_{2}\frac{\partial j_{1}}{\partial z}=0.$$

Here, the skew-symmetric matrix N is called the Poisson matrix.

On a three-dimensional space, a skew-symmetric Poisson matrix N can be isomorphic to a vector J, and the map is as follows:


(2.15) $$J=(j_{1},j_{2},j_{3})↔N=\left(\begin{array}{ccc} 0 & -j_{3} & j_{2}\\ j_{3} & 0 & -j_{1}\\ -j_{2} & j_{1} & 0 \end{array}\right),$$

where J is called the Poisson vector. The establishment of equation (2.15) is owing to the identity NB=J×B with arbitrary vector B.

For a three-dimensional nonlinear system given in equation (2.11), if there is a generalized Poisson bracket {⋅,⋅} (corresponding to Poisson matrix N) and a real-valued function H, the system can be written in the form of the Hamiltonian equation


(2.16) $$\dot{u}=N\nabla H\;or\;\dot{u}=\{u,H\},$$

then the system is considered to have a generalized Hamiltonian structure, and H is called Hamiltonian function. The Hamiltonian function of a system can usually be obtained through the first integral.

Secondly, the following introduces two theorems about the generalized Hamiltonian system.

***Theorem 3******.** If the three-dimensional nonlinear system given in* *equation (2.11)* *has a time-independent first integral and there is a function* M *satisfying* divMF(u)=0*, then* *equation (2.11)* *has a generalized Hamiltonian structure and is a bi-Hamiltonian system* [56].

*Actually, the function* M *in theorem 3 is called Jacobi’s last multiplier and satisfies*


(2.17) $$J=\frac{1}{M}\nabla C,$$

*where* J *is the Poisson vector and* C *is a Casimir function of vector* J*.* *Equation (2.17)* *is the general solution of Jacobi identity (**equation (2.14)**), one of the conditions that the generalized Poisson bracket needs to satisfy. Detailed content can be found in Esen et al.* [44].

***Theorem 4.*** *Suppose* $x_{e}$ *is the equilibrium point of a three-dimensional generalized Hamiltonian system given in* *equation (2.16)* *and a regular point of Poisson matrix* N(u)*, and the rank of* $N(x_{e})$ *is 2.* [57,58]. *If the linearized system corresponding to the three-dimensional generalized Hamiltonian system at equilibrium point* $x_{e}$ *has non-zero eigenvalues, it must belong to one of the following situations:*

*(1)* $\lambda_{1}=0,\lambda_{2,3}=\pm \mu (\mu >0)$;

*(2)* $\lambda_{1}=0,\lambda_{2,3}=\pm \mu i(\mu >0,i=\sqrt{-1})$.

*In case (1),* $x_{e}$ *is the hyperbolic saddle point that restricts the system on the symplectic leaf and is therefore unstable. In case (2), the equilibrium point* $x_{e}$ *is a nonlinear centre of the constrained system on the symplectic leaf, and near* $x_{e}$*, the orbits are closed within the symplectic leaf.*

Finally, the 2×2×2 asymmetric evolutionary game (equation (2.3)) is analysed by generalized Hamiltonian system theory.

***Theorem 5.*** *When the system given in* *equation (2.3)* *satisfies the condition given in* *equation (2.9)**, the system has a time-independent first integral, that is,*


(2.18) $$c_{12}\ln(1-x)-c_{22}\ln x-a_{21}\ln(1-z)+a_{22}\ln z=c,$$

where c is a constant.

*Proof*. From the proof process of theorem 2, when equation (2.9) holds, i.e. $a_{12}c_{22}=a_{22}c_{21}$, we have


$$\frac{1}{a_{22}}((a_{12}-a_{22})y+a_{22})=\frac{1}{c_{22}}((c_{21}-c_{22})y+c_{22}),$$

further,


$$\begin{array}{ll} \frac{dx}{dz} & =\frac{x\left(1-x\right)f\left(y,z\right)}{z\left(1-z\right)h\left(x,y\right)}\\ & =\frac{\frac{1}{a_{22}}\left(\left(a_{12}-a_{22}\right)y+a_{22}\right)\left(\left(a_{21}-a_{22}\right)z+a_{22}\right)x\left(1-x\right)}{\frac{1}{c_{22}}\left(\left(c_{12}-c_{22}\right)x+c_{22}\right)\left(\left(c_{21}-c_{22}\right)y+c_{22}\right)z\left(1-z\right)}\\ & =\frac{\left(\left(a_{21}-a_{22}\right)z+a_{22}\right)x\left(1-x\right)}{\left(\left(c_{12}-c_{22}\right)x+c_{22}\right)z\left(1-z\right)}, \end{array}$$

thus,


$$\int \frac{(c_{12}-c_{22})x+c_{22}}{x(1-x)}dx=\int \frac{(a_{21}-a_{22})z+a_{22}}{z(1-z)}dx,$$

further,


$$-c_{12}\ln(1-x)+c_{22}\ln x=-a_{21}\ln(1-z)+a_{22}\ln z.$$

Therefore, the time-independent first integral is


$$c_{12}\ln(1-x)-c_{22}\ln x-a_{21}\ln(1-z)+a_{22}\ln z=c.$$

∎

***Theorem 6.*** *When the system given in* *equation (2.3)* *satisfies the condition given in* *equation (2.9)**, the system has a generalized Hamiltonian structure and is a bi-Hamiltonian system*.

*Proof*. From theorem 5, the system given in equation (2.3) has a time-independent first integral, i.e.


$$c_{12}\ln(1-x)-c_{22}\ln x-a_{21}\ln(1-z)+a_{22}\ln z=c.$$

Record the real-valued function


(2.19) $$M=\frac{1}{x(1-x)y(1-y)z(1-z)},$$

it is easy to prove that


$$divM\overset{˙}{u}=0$$

with u=(x,y,z).

Thus, according to theorem 3, we can draw a conclusion that the system has a generalized Hamiltonian structure and is a bi-Hamiltonian system when equation (2.3) satisfies the condition given in equation (2.9).

∎

***Corollary 1*.** *When the system given in* *equation (2.3)* *satisfies the condition given in* *equation (2.9)**, the generalized Hamiltonian system given in* *equation (2.3)* *has a Poisson vector* J*, making*


(2.20) $$\dot{u}=J\times \nabla H,$$

*where,* u=(x,y,z), *Hamiltonian function*


$$H=c_{12}\ln(1-x)-c_{22}\ln x-a_{21}\ln(1-z)+a_{22}\ln z,$$

*and Poisson vector*


$$\begin{array}{c} J=\left(-\frac{z\left(1\;-\;z\right)\left(\frac{1}{b_{22}}y\left(1\;-\;y\right)\left(\left(b_{21}\;-\;b_{22}\right)x\;+\;b_{22}\right)\left(\left(b_{12}\;-\;b_{22}\right)z\;+\;b_{22}\right)\;+\;\frac{\left(c_{12}\;-\;c_{22}\right)x\;+\;c_{22}}{x\left(1\;-\;x\right)}\right)}{\left(a_{21}\;-\;a_{22}\right)z\;+\;a_{22}},\;\frac{x\left(1\;-\;x\right)z\left(1\;-\;z\right)\left(\left(a_{12}\;-\;a_{22}\right)y\;+\;a_{22}\right)}{a_{22}},1\right). \end{array}$$

*Proof*. According to the first integral in theorem 5, it is known that a Hamiltonian function of the system given in equation (2.3) is


$$H=c_{12}\ln(1-x)-c_{22}\ln x-a_{21}\ln(1-z)+a_{22}\ln z.$$

Furthermore, we have the first-order variational


$$\nabla H=\left(-\frac{\left(c_{12}\;-\;c_{22}\right)x\;+\;c_{22}}{x\left(1\;-\;x\right)},0,\frac{\left(a_{21}\;-\;a_{22}\right)z\;+\;a_{22}}{z\left(1\;-\;z\right)}\right).$$

Suppose that the expression of the Poisson vector is


J=(α,β,γ),

we have


$$\begin{array}{l} \left(\alpha ,\beta ,\gamma \right)\times \left(-\frac{\left(c_{12}\;-\;c_{22}\right)x\;+\;c_{22}}{x\left(1\;-\;x\right)},0,\frac{\left(a_{21}\;-\;a_{22}\right)z\;+\;a_{22}}{z\left(1\;-\;z\right)}\right)\\ =\left(x\left(1\;-\;x\right)f\left(y,z\right),y\left(1\;-\;y\right)g\left(z,x\right),z\left(1\;-\;z\right)h\left(x,y\right)\right), \end{array}$$

thus,


$$\begin{array}{l} \frac{(a_{21}-a_{22})z\;+\;a_{22}}{z(1\;-\;z)}\beta =x(1\;-\;x)[\frac{1}{a_{22}}((a_{12}-a_{22})y\;+\;a_{22})((a_{21}\;-\;a_{22})z+a_{22})],\\ -\frac{(a_{21}-a_{22})z\;+\;a_{22}}{z(1\;-\;z)}\alpha -\frac{(c_{12}\;-\;c_{22})x\;+\;c_{22}}{x(1\;-\;x)}\gamma =y(1\;-\;y)[\frac{1}{b_{22}}((b_{12}\;-\;b_{22})z\;+\;b_{22})((b_{21}\;-\;b_{22})x\;+\;b_{22})],\\ \frac{(c_{12}-c_{22})x\;+\;c_{22}}{x(1-x)}\beta =z(1-z)[\frac{1}{c_{22}}((c_{12}-c_{22})x+c_{22})((c_{21}-c_{22})y+c_{22})], \end{array}$$

it follows that


$$\beta =\frac{x\left(1\;-\;x\right)z\left(1\;-\;z\right)\left(\left(a_{12}\;-\;a_{22}\right)y\;+\;a_{22}\right)}{a_{22}}=\frac{x\left(1\;-\;x\right)z\left(1\;-\;z\right)\left(\left(c_{21}\;-\;c_{22}\right)y\;+\;c_{22}\right)}{c_{22}}.$$

It obviously holds from equation (2.9). Let γ=1, we have


$$\alpha =-\frac{z\left(1\;-\;z\right)\left(\frac{1}{b_{22}}y\left(1\;-\;y\right)\left(\left(b_{21}\;-\;b_{22}\right)x\;+\;b_{22}\right)\left(\left(b_{12}\;-\;b_{22}\right)z\;+\;b_{22}\right)\;+\;\frac{\left(c_{12}\;-\;c_{22}\right)x\;+\;c_{22}}{x\left(1\;-\;x\right)}\right)}{\left(a_{21}\;-\;a_{22}\right)z\;+\;a_{22}}.$$

Thus, the Poisson vector is


$$J=\left(-\frac{z\left(1\;-\;z\right)\left(\frac{1}{b_{22}}y\left(1\;-\;y\right)\left(\left(b_{21}-b_{22}\right)x\;+\;b_{22}\right)\left(\left(b_{12}\;-\;b_{22}\right)z\;+\;b_{22}\right)\;+\;\frac{\left(c_{12}\;-\;c_{22}\right)x\;+\;c_{22}}{x\left(1\;-\;x\right)}\right)}{\left(a_{21}\;-\;a_{22}\right)z\;+\;a_{22}},\frac{x\left(1\;-\;x\right)z\left(1\;-\;z\right)\left(\left(a_{12}\;-\;a_{22}\right)y\;+\;a_{22}\right)}{a_{22}},1\right).$$

∎

The Hamiltonian function H is obtained by solving the first integral (theorem 5). By assuming $a_{12}c_{22}=a_{22}c_{21}$, it can be inferred that the linear function about y in f(y,z) is proportional to that in h(x,y) (see the proof process of theorem 2). Consequently, the ratio between dx and dz can be expressed separably using variables x and z, forming a kind of symmetry and integrability. Furthermore, when integrating both dx and dz simultaneously, the first integral and Hamiltonian function of the system can be derived.

Besides, the Poisson vector obtained from corollary 1 is not unique. For instance, let α=0, we have


$$\gamma =-\frac{y\left(1\;-\;y\right)x\left(1\;-\;x\right)\left(\left(b_{21}\;-\;b_{22}\right)x\;+\;b_{22}\right)\left(\left(b_{12}\;-\;b_{22}\right)z\;+\;b_{22}\right)}{b_{22}\left(\left(c_{12}\;-\;c_{22}\right)x\;+\;c_{22}\right)},$$

thus, the Poisson vector is


$$J=\left(0,\frac{x\left(1\;-\;x\right)z\left(1\;-\;z\right)\left(\left(a_{12}\;-\;a_{22}\right)y\;+\;a_{22}\right)}{a_{22}},-\frac{y\left(1\;-\;y\right)x\left(1\;-\;x\right)\left(\left(b_{21}\;-\;b_{22}\right)x\;+\;b_{22}\right)\left(\left(b_{12}\;-\;b_{22}\right)z\;+\;b_{22}\right)}{b_{22}\left(\left(c_{12}\;-\;c_{22}\right)x\;+\;c_{22}\right)}\right);$$

let γ=0, we have


$$\alpha =-\frac{y\left(1\;-\;y\right)z\left(1\;-\;z\right)\left(\left(b_{21}\;-\;b_{22}\right)x\;+\;b_{22}\right)\left(\left(b_{12}\;-\;b_{22}\right)z\;+\;b_{22}\right)}{b_{22}\left(\left(a_{21}\;-\;a_{22}\right)z\;+\;a_{22}\right)},$$

thus, the Poisson vector is


$$J=\left(-\frac{y\left(1\;-\;y\right)z\left(1\;-\;z\right)\left(\left(b_{21}\;-\;b_{22}\right)x\;+\;b_{22}\right)\left(\left(b_{12}\;-\;b_{22}\right)z\;+\;b_{22}\right)}{b_{22}\left(\left(a_{21}\;-\;a_{22}\right)z\;+\;a_{22}\right)},\frac{x\left(1\;-\;x\right)z\left(1\;-\;z\right)\left(\left(a_{12}\;-\;a_{22}\right)y\;+\;a_{22}\right)}{a_{22}},0\right).$$

### 2.3. The stability of interior equilibrium points

When the system given in equation (2.3) satisfies the conditions given in equations (2.9) and (2.10) from theorem 2, for $\forall \tilde{x}\in (0,1)$, the point $\left(\tilde{x},\frac{a_{22}}{a_{22}-a_{12}},\frac{b_{22}}{b_{22}-b_{12}}\right)$ is an interior equilibrium point. Taking these points as examples, we will discuss their stability by means of generalized Hamiltonian system theory. Before that, a few lemmas are given first.

***Lemma 1.*** *When the system given in* *equation (2.3)* *satisfies the conditions given in* *equations (2.9)* *and* *(2.10)*, *for* $\forall \tilde{x}\in (0,1)$*, the interior equilibrium point*


$$\left(x^{*},y^{*},z^{*})=(\tilde{x},\frac{a_{22}}{a_{22}-a_{12}},\frac{b_{22}}{b_{22}-b_{12}}\right)$$

*is a regular point of the Poisson matrix* N *corresponding to the Poisson vector* J *in* *equation (2.20)**, and the rank of* $N(x^{*},y^{*},z^{*})$ *is 2.*

*Proof*. For $\forall \tilde{x}\in (0,1)$, substituting the interior equilibrium point


$$\left(x^{*},y^{*},z^{*}\right)=\left(\overset{˜}{x},\frac{a_{22}}{a_{22}\;-\;a_{12}},\frac{b_{22}}{b_{22}\;-\;b_{12}}\right)$$

into Poisson vector


$$\begin{array}{c} J=\left(-\frac{z\left(1-z\right)\left(\frac{1}{b_{22}}y\left(1-y\right)\left(\left(b_{21}-b_{22}\right)x+b_{22}\right)\left(\left(b_{12}-b_{22}\right)z+b_{22}\right)+\frac{\left(c_{12}-c_{22}\right)x+c_{22}}{x\left(1-x\right)}\right)}{\left(a_{21}-a_{22}\right)z+a_{22}},\;\frac{x\left(1-x\right)z\left(1-z\right)\left(\left(a_{12}-a_{22}\right)y+a_{22}\right)}{a_{22}},1\right), \end{array}$$

we have


$$J\left(x^{*},y^{*},z^{*}\right)=\left(\frac{b_{12}b_{22}\left(\left(c_{12}-c_{22}\right)\overset{˜}{x}+c_{22}\right)}{\left(a_{21}b_{22}-a_{22}b_{12}\right)\left(b_{22}-b_{12}\right)\overset{˜}{x}\left(1-\overset{˜}{x}\right)},0,1\right).$$

When $\tilde{x}=\frac{c_{22}}{c_{22}-c_{12}}$, $J(x^{*},y^{*},z^{*})=(0,0,1)$, and the corresponding Poisson matrix is


$$N=\left(\begin{array}{ccc} 0 & -j_{3} & j_{2}\\ j_{3} & 0 & -j_{1}\\ -j_{2} & j_{1} & 0 \end{array}\right)=\left(\begin{array}{ccc} 0 & -1 & 0\\ 1 & 0 & 0\\ 0 & 0 & 0 \end{array}\right),$$

thus, the rank of


$$N(x^{*},y^{*},z^{*})=N(\tilde{x},\frac{a_{22}}{a_{22}-a_{12}},\frac{b_{22}}{b_{22}-b_{12}}),\;\;\tilde{x}=\frac{c_{22}}{c_{22}-c_{12}}$$

is 2.

When $\tilde{x}\in (0,1)$ and $\tilde{x}\neq \frac{c_{22}}{c_{22}-c_{12}}$, $J(x^{*},y^{*},z^{*})=(j_{1},0,1)$ with $j_{1}\neq 0$, and the corresponding Poisson matrix is


$$N=\left(\begin{array}{ccc} 0 & -j_{3} & j_{2}\\ j_{3} & 0 & -j_{1}\\ -j_{2} & j_{1} & 0 \end{array}\right)=\left(\begin{array}{ccc} 0 & -1 & 0\\ 1 & 0 & -j_{1}\\ 0 & j_{1} & 0 \end{array}\right),$$

thus, the rank of


$$N(x^{*},y^{*},z^{*})=N(\tilde{x},\frac{a_{22}}{a_{22}-a_{12}},\frac{b_{22}}{b_{22}-b_{12}}),\;\;\tilde{x}\in (0,\frac{c_{22}}{c_{22}-c_{12}})\cup (\frac{c_{22}}{c_{22}-c_{12}},1)$$

is 2 as well.

To sum up, for $\forall \tilde{x}\in (0,1)$, the interior equilibrium point $\left(\tilde{x},\frac{a_{22}}{a_{22}-a_{12}},\frac{b_{22}}{b_{22}-b_{12}}\right)$ is a regular point of the Poisson matrix N corresponding to the Poisson vector J in equation (2.20), and the rank of $N(\tilde{x},\frac{a_{22}}{a_{22}-a_{12}},\frac{b_{22}}{b_{22}-b_{12}})$ is 2.

∎

***Lemma 2******.** When the system given in* *equation (2.3)* *satisfies the conditions given in* *equations (2.9)* *and* *(2.10)*, *for* $\forall \tilde{x}\in (0,1)$*, the linearized system corresponding to the system given in* *equation (2.3)* *at the interior equilibrium point* $\left(\tilde{x},\frac{a_{22}}{a_{22}-a_{12}},\frac{b_{22}}{b_{22}-b_{12}}\right)$ *has a zero eigenvalue.*

*Proof*. Let


$$F(x,y,z)=\dot{x},\;G(x,y,z)=\dot{y},\;H(x,y,z)=\dot{z},$$

we have the Jacobi matrix about F(x,y,z), G(x,y,z) and H(x,y,z), that is, equation (2.6).

From $a_{12}c_{22}=a_{22}c_{21}$ in equation (2.9), it can be obtained that


$$\frac{a_{22}}{a_{22}-a_{12}}=\frac{c_{22}}{c_{22}-c_{21}}.$$

Furthermore, for $\forall \tilde{x}\in (0,1)$, the Jacobi matrix at the interior equilibrium point $\left(\tilde{x},\frac{a_{22}}{a_{22}-a_{12}},\frac{b_{22}}{b_{22}-b_{12}}\right)$ is


$$Jacobi{|}_{\left(\tilde{x},\frac{a_{22}}{a_{22}-a_{12}},\frac{b_{22}}{b_{22}-b_{12}}\right)}=\left(\begin{array}{ccc} 0 & \frac{\tilde{x}(1-\tilde{x})(a_{12}-a_{22})(b_{22}a_{21}-a_{22}b_{12})}{a_{22}(b_{22}-b_{12})} & 0\\ 0 & 0 & -\frac{c_{22}c_{21}(b_{12}-b_{22})((b_{21}-b_{22})\tilde{x}+b_{22})}{b_{22}(c_{22}-c_{21}{)}^{2}}\\ 0 & -\frac{b_{22}b_{12}(c_{21}-c_{22})((c_{12}-c_{22})\tilde{x}+c_{22})}{c_{22}(b_{22}-b_{12}{)}^{2}} & 0 \end{array}\right),$$

thus, we have eigenvalues


$$\lambda_{1}=0,\;\lambda_{2,3}=\pm \frac{\sqrt{b_{12}c_{21}\left(b_{12}-b_{22}\right)\left(\left(b_{21}-b_{22}\right)\overset{˜}{x}+b_{22}\right)\left(c_{21}-c_{22}\right)\left(\left(c_{12}-c_{22}\right)\overset{˜}{x}+c_{22}\right)}}{\left(c_{22}-c_{21}\right)\left(b_{22}-b_{12}\right)}.$$

∎

***Lemma 3*.** *Let*


$$E=\left(\begin{array}{c} 0\\ 1 \end{array}\right)\;\;\;\;and\;\;\;\;\;\overset{˜}{X}=\left(\begin{array}{c} \overset{˜}{x}\\ 1-\overset{˜}{x} \end{array}\right),$$

*where* $\tilde{x}\in (0,1)$. *Furthermore, assume that the system given in* *equation (2.3)* *satisfies the conditions given in* *equations (2.9)* *and* *(2.10)**. If the inequality*


(2.21) $$E^{T}M_{B}\overset{˜}{X}{\overset{˜}{X}}^{T}M_{C}E<0$$

*holds, then it follows that*


$$b_{12}c_{21}\left(b_{12}-b_{22}\right)\left(\left(b_{21}-b_{22}\right)\overset{˜}{x}+b_{22}\right)\left(c_{21}-c_{22}\right)\left(\left(c_{12}-c_{22}\right)\overset{˜}{x}+c_{22}\right)<0.$$

*Here, the symbol* T *represents transposition.*

*Proof*. From the condition given in equation (2.10) holds, it can be drawn that $a_{12}a_{22}<0$ and $b_{12}b_{22}<0$, combining $a_{12}c_{22}=a_{22}c_{21}$ in equation (2.9), we have $c_{21}c_{22}<0$. Furthermore, it follows that


$$\begin{array}{c} b_{12}(b_{12}-b_{22})=b_{12}^{2}-b_{12}b_{22}>0,\\ c_{21}(c_{21}-c_{22})=c_{21}^{2}-c_{21}c_{22}>0. \end{array}$$

Calculating


$$E^{T}M_{B}\overset{˜}{X}=\left(0,1\right)\left(\begin{array}{cc} b_{11} & b_{12}\\ b_{21} & b_{22} \end{array}\right)\left(\begin{array}{c} \overset{˜}{x}\\ 1-\overset{˜}{x} \end{array}\right)=\left(b_{21}-b_{22}\right)\overset{˜}{x}+b_{22}$$

and


$${\tilde{X}}^{T}M_{C}E=(\tilde{x},1-\tilde{x})\left(\begin{array}{cc} c_{11} & c_{12}\\ c_{21} & c_{22} \end{array}\right)\left(\begin{array}{c} 0\\ 1 \end{array}\right)=(c_{12}-c_{22})\tilde{x}+c_{22},$$

we have


$$E^{T}M_{B}\overset{˜}{X}{\overset{˜}{X}}^{T}M_{C}E=\left(\left(b_{21}-b_{22}\right)\overset{˜}{x}+b_{22}\right)\left(\left(c_{12}-c_{22}\right)\overset{˜}{x}+c_{22}\right).$$

To sum up, when $E^{T}M_{B}\tilde{X}{\tilde{X}}^{T}M_{C}E<0$, it follows that


$$b_{12}c_{21}\left(b_{12}-b_{22}\right)\left(\left(b_{21}-b_{22}\right)\overset{˜}{x}+b_{22}\right)\left(c_{21}-c_{22}\right)\left(\left(c_{12}-c_{22}\right)\overset{˜}{x}+c_{22}\right)<0.$$

∎

When $\tilde{x}=\frac{c_{22}}{c_{22}-c_{12}}$, obviously, $E^{T}M_{B}\tilde{X}{\tilde{X}}^{T}M_{C}E=0$. Hence, the stability judgement method here is not applicable to discuss the stability of the interior equilibrium point $\left(\frac{c_{22}}{c_{22}-c_{12}},\frac{a_{22}}{a_{22}-a_{12}},\frac{b_{22}}{b_{22}-b_{12}}\right)$; similarly, is not applicable to discuss the stability of the interior equilibrium point $\left(\frac{b_{22}}{b_{22}-b_{21}},\frac{c_{22}}{c_{22}-c_{21}},\frac{a_{22}}{a_{22}-a_{21}}\right)$ as well.

***Theorem 7.*** *Let*


$$E=\left(\begin{array}{c} 0\\ 1 \end{array}\right)\;\;\;\;and\;\;\;\;\;\overset{˜}{X}=\left(\begin{array}{c} \overset{˜}{x}\\ 1-\overset{˜}{x} \end{array}\right),$$

*where* $\tilde{x}\in (0,1)$*. Furthermore, assume that the system given in* *equation (2.3)* *satisfies the conditions given in* *equations (2.9)* *and* *(2.10)**. If the inequality*


$$E^{T}M_{B}\overset{˜}{X}{\overset{˜}{X}}^{T}M_{C}E<0$$

*holds as the interior equilibrium point*


$$(x^{*},y^{*},z^{*})=(\tilde{x},\frac{a_{22}}{a_{22}-a_{12}},\frac{b_{22}}{b_{22}-b_{12}}),$$

*then this point is a nonlinear centre and thus Lyapunov stable.*

*Proof*. Because the system given in equation (2.3) satisfies the condition given in equation (2.9), according to theorem 6, equation (2.3) is a three-dimensional generalized Hamiltonian system.

Furthermore, because the system given in equation (2.3) satisfies the condition given in equation (2.10), from theorem 2, for $\forall \tilde{x}\in (0,1)$, the point $\left(\tilde{x},\frac{a_{22}}{a_{22}-a_{12}},\frac{b_{22}}{b_{22}-b_{12}}\right)$ is an interior equilibrium point.

From lemma 1, for $\forall \tilde{x}\in (0,1)$, the interior equilibrium point $\left(\tilde{x},\frac{a_{22}}{a_{22}-a_{12}},\frac{b_{22}}{b_{22}-b_{12}}\right)$ is a regular point of the Poisson matrix N corresponding to the Poisson vector J in equation (2.20), and the rank of $N(\tilde{x},\frac{a_{22}}{a_{22}-a_{12}},\frac{b_{22}}{b_{22}-b_{12}})$ is 2.

From lemma 2, the eigenvalues of the linearized system corresponding to the system given in equation (2.3) at the interior equilibrium point $\left(\tilde{x},\frac{a_{22}}{a_{22}-a_{12}},\frac{b_{22}}{b_{22}-b_{12}}\right)$ is


$$\lambda_{1}=0,\lambda_{2,3}=\pm \frac{\sqrt{b_{12}c_{21}\left(b_{12}-b_{22}\right)\left(\left(b_{21}-b_{22}\right)\overset{˜}{x}+b_{22}\right)\left(c_{21}-c_{22}\right)\left(\left(c_{12}-c_{22}\right)\overset{˜}{x}+c_{22}\right)}}{\left(c_{22}-c_{21}\right)\left(b_{22}-b_{12}\right)}.$$

From lemma 3, when the inequality


$$E^{T}M_{B}\tilde{X}{\tilde{X}}^{T}M_{C}E<0$$

holds, it follows that


$$b_{12}c_{21}\left(b_{12}-b_{22}\right)\left(\left(b_{21}-b_{22}\right)\overset{˜}{x}+b_{22}\right)\left(c_{21}-c_{22}\right)\left(\left(c_{12}-c_{22}\right)\overset{˜}{x}+c_{22}\right)<0,$$

thus,


$$\lambda_{2,3}=\pm \mu i(\mu >0,i=\sqrt{-1}).$$

Finally, according to theorem 4, the interior equilibrium point $\left(\tilde{x},\frac{a_{22}}{a_{22}-a_{12}},\frac{b_{22}}{b_{22}-b_{12}}\right)$ of the generalized Hamiltonian system given in equation (2.3) is a nonlinear centre and thus Lyapunov stable.

∎

### 2.4. Brief summary

Based on the above-mentioned analysis, the conditions that the parameters need to satisfy when six types of points are interior equilibrium points and are stable interior equilibrium points can be summarized, which are presented in the form of a table, as shown in table 2.

**Table 2.** The judgement conditions of stable interior equilibrium points.^a^

points	the conditions as interior equilibrium points	the conditions as stable interior equilibrium points^b^^c^
$\left(\tilde{x},\frac{a_{22}}{a_{22}-a_{12}},\frac{b_{22}}{b_{22}-b_{12}}\right)$, $\tilde{x}\in (0,1)$	$a_{12}a_{22}<0$, $b_{12}b_{22}<0$, $a_{12}c_{22}=a_{22}c_{21}$	$E^{T}M_{B}\tilde{X}{\tilde{X}}^{T}M_{C}E<0$
$\left(\frac{c_{22}}{c_{22}-c_{12}},\tilde{y},\frac{b_{22}}{b_{22}-b_{12}}\right)$, $\tilde{y}\in (0,1)$	$b_{12}b_{22}<0$, $c_{12}c_{22}<0$, $b_{12}a_{22}=b_{22}a_{21}$	$E^{T}M_{C}\tilde{Y}{\tilde{Y}}^{T}M_{A}E<0$
$\left(\frac{c_{22}}{c_{22}-c_{12}},\frac{a_{22}}{a_{22}-a_{12}},\tilde{z}\right)$, $\tilde{z}\in (0,1)$	$c_{12}c_{22}<0$, $a_{12}a_{22}<0$, $c_{12}b_{22}=c_{22}b_{21}$	$E^{T}M_{A}\tilde{Z}{\tilde{Z}}^{T}M_{B}E<0$
$\left(\tilde{x},\frac{c_{22}}{c_{22}-c_{21}},\frac{a_{22}}{a_{22}-a_{21}}\right)$, $\tilde{x}\in (0,1)$	$c_{21}c_{22}<0$, $a_{21}a_{22}<0$, $b_{12}a_{22}=b_{22}a_{21}$	$E^{T}M_{B}\tilde{X}{\tilde{X}}^{T}M_{C}E<0$
$\left(\frac{b_{22}}{b_{22}-b_{21}},\tilde{y},\frac{a_{22}}{a_{22}-a_{21}}\right)$, $\tilde{y}\in (0,1)$	$a_{21}a_{22}<0$, $b_{21}b_{22}<0$, $c_{12}b_{22}=c_{22}b_{21}$	$E^{T}M_{C}\tilde{Y}{\tilde{Y}}^{T}M_{A}E<0$
$\left(\frac{b_{22}}{b_{22}-b_{21}},\frac{c_{22}}{c_{22}-c_{21}},\tilde{z}\right)$, $\tilde{z}\in (0,1)$	$b_{21}b_{22}<0$, $c_{21}c_{22}<0$, $a_{12}c_{22}=a_{22}c_{21}$	$E^{T}M_{A}\tilde{Z}{\tilde{Z}}^{T}M_{B}E<0$

^a^ Here is to judge interior equilibrium points of the system given in equation (2.3), that is, the payoff difference matrices in equation (2.1) satisfy $|M_{A}|=0$, $|M_{B}|=0$, $|M_{C}|=0$.

^b^ Here, $E={\left(0,1\right)}^{T}$, $\tilde{X}={\left(\tilde{x},1-\tilde{x}\right)}^{T}$, $\tilde{Y}={\left(\tilde{y},1-\tilde{y}\right)}^{T}$, $\tilde{Z}={\left(\tilde{z},1-\tilde{z}\right)}^{T}$.

^c^ There is a hierarchical progressive relationship between the two conditions from left to right, that is, only when the conditions as interior equilibrium points are met can we continue to judge whether they are stable interior equilibrium points.

When there are stable interior equilibrium points in the 2×2×2 asymmetric games evolution system, under some initial conditions, there must be closed curves centred on these stable interior equilibrium points in the phase space. It reflects that the resulting state of the system is that two strategies coexist in each population, and the proportion of strategies fluctuates over time, i.e. there is an endless oscillation.

### 2.5. Example

In order to gain a more intuitive understanding of the conclusions drawn here, the following is an example to illustrate.

Assume that the payoff difference matrices corresponding to three populations are


(2.22) $$M_{A}=\left(\begin{array}{cc} 1 & 1\\ -1 & -1 \end{array}\right),M_{B}=\left(\begin{array}{cc} -1 & 1\\ 1 & -1 \end{array}\right),M_{C}=\left(\begin{array}{cc} 1 & -1\\ -1 & 1 \end{array}\right).$$

By calculating the determinant, one can know that $|M_{A}|=0$, $|M_{B}|=0$ and $|M_{C}|=0$. Thus, the system described by equation (2.22) is a class of 2×2×2 asymmetric games considered in this paper.

Because $a_{12}c_{22}=1\times 1=1$ and $a_{22}c_{21}=-1\times (-1)=1$, thus, we have $a_{12}c_{22}=a_{22}c_{21}$, that is, the condition given in equation (2.9) is satisfied.

Furthermore, because $a_{12}a_{22}=1\times (-1)=-1<0$ and $b_{12}b_{22}=1\times (-1)=-1<0$, according to theorem 2, for $\forall \tilde{x}\in (0,1)$, the point


$$\left(\overset{˜}{x},\frac{a_{22}}{a_{22}-a_{12}},\frac{b_{22}}{b_{22}-b_{12}}\right)=\left(\overset{˜}{x},\frac{-1}{-1-1},\frac{-1}{-1-1}\right)=\left(\overset{˜}{x},\frac{1}{2},\frac{1}{2}\right)$$

is an interior equilibrium point of the system given in equation (2.22).

Record


$$E=\left(\begin{array}{c} 0\\ 1 \end{array}\right)\;\;\;\;and\;\;\;\;\;\overset{˜}{X}=\left(\begin{array}{c} \overset{˜}{x}\\ 1-\overset{˜}{x} \end{array}\right)$$

then


$$E^{T}M_{B}\overset{˜}{X}{\overset{˜}{X}}^{T}M_{C}E=\left(0,1\right)\left(\begin{array}{cc} -1 & 1\\ 1 & -1 \end{array}\right)\left(\begin{array}{c} \overset{˜}{x}\\ 1-\overset{˜}{x} \end{array}\right)\left(\overset{˜}{x},1-\overset{˜}{x}\right)\left(\begin{array}{cc} 1 & -1\\ -1 & 1 \end{array}\right)\left(\begin{array}{c} 0\\ 1 \end{array}\right)=-{\left(2\overset{˜}{x}-1\right)}^{2}.$$

Furthermore, according to theorem 7, interior equilibrium points


$$\left(x^{*},y^{*},z^{*})=(\tilde{x},\frac{1}{2},\frac{1}{2}),\;\tilde{x}\in (0,\frac{1}{2})\cup (\frac{1}{2},1\right)$$

are stable.

In fact, based on the analysis in this paper, more information about the properties of the system given in equation (2.22) can be obtained.

According to proposition 1, the system given in equation (2.22) is


(2.23) $$\{\begin{array}{cc} \dot{x} & =x(1-x)(2y-1)\\ \dot{y} & =-y(1-y)(2x-1)(2z-1)\\ \dot{z} & =z(1-z)(2x-1)(2y-1) \end{array}.$$

From theorem 6 and corollary 1, the system given in equation (2.23) has a generalized Hamiltonian structure with Hamiltonian function H,


(2.24) H=−ln(1−x)−lnx+ln(1−z)−lnz,

and Poisson vector J. When the expression of Poisson vector J is


$$J=\left(0,\frac{x\left(1-x\right)z\left(1-z\right)\left(\left(a_{12}-a_{22}\right)y+a_{22}\right)}{a_{22}},-\frac{y\left(1-y\right)x\left(1-x\right)\left(\left(b_{21}-b_{22}\right)x+b_{22}\right)\left(\left(b_{12}-b_{22}\right)z+b_{22}\right)}{b_{22}\left(\left(c_{12}-c_{22}\right)x+c_{22}\right)}\right),$$

we have


(2.25) J=(0,−x(1−x)z(1−z)(2y−1),−x(1−x)y(1−y)(2z−1)).

Furthermore, equation (2.23) can be expressed using equations (2.24) and (2.25), that is,


$$\dot{u}=J\times \nabla H,$$

here u=(x,y,z).

The explicit expression of the Casimir function (equation (2.17)) mentioned in this paper is generally not easy to obtain [57]; however, in this example, it can be figured out that


(2.26) C=ln(1−y)+lny+ln(1−z)+lnz,

which satisfies equation (2.17), i.e.


$$J=\frac{1}{M}\nabla C,$$

where $M=\frac{1}{x\left(1-x\right)y\left(1-y\right)z\left(1-z\right)}.$

Therefore, the system given in equation (2.23) can also be represented by Hamiltonian function H and Casimir function C, that is,


(2.27) $$\dot{u}=\frac{1}{M}\nabla C\times \nabla H,$$

where u=(x,y,z).

**Figure 1.**
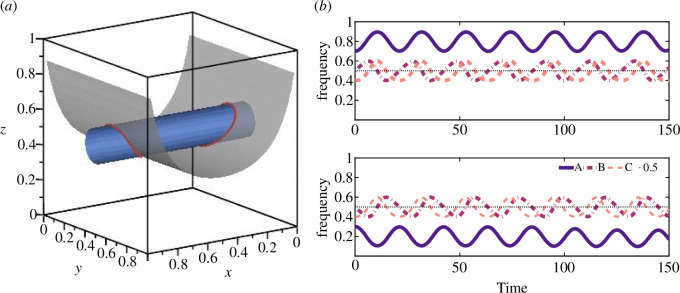
Initial values are (0.7,0.5,0.4) and (0.3,0.5,0.4). (*a*) Invariant surfaces in spatial phase diagram and (*b*) the proportion of strategies over time diagrams. In *(a)*, the expressions of the two invariant surfaces are −ln(1−x)−lnx+ln(1−z)−lnz=1.96611285 and ln(1−y)+lny+ln(1−z)+lnz=−2.81341071, respectively. In effect, the intersection line of two invariant surfaces in *(a)* forms two closed curves where the points evolve over time as shown in *(b)*.

Actually, both the Hamiltonian function and the Casimir function are conserved quantities of three-dimensional autonomous differential systems; furthermore, we have


(2.28) $$H=-ln\left(1-x\right)-lnx+ln\left(1-z\right)-lnz=c_{1}$$

and


(2.29) $$C=\ln(1-y)+\ln y+\ln(1-z)+\ln z=c_{2},$$

where both $c_{1}$ and $c_{2}$ are constants determined by initial conditions. Hamiltonian function and Casimir function describe two invariant surfaces, respectively, and the intersection line of the two surfaces is closed orbits surrounding stable interior equilibrium points. Two initial values are taken to illustrate this phenomenon, as shown in figure 1.

When initial values of the system given in equation (2.23) are set to (0.7,0.5,0.4) and (0.3,0.5,0.4), the spatial phase diagram and time evolution diagram are shown in figure 1. Substituting the initial value (0.7,0.5,0.4) into equations (2.28) and (2.29), respectively, it can be drawn that $c_{1}=1.96611285$ and $c_{2}=-2.81341071$. Thus, the two invariant surfaces are −ln(1−x)−lnx+ln(1−z)−lnz=1.96611285 and ln(1−y)+lny+ln(1−z)+lnz=−2.81341071, respectively. Besides, the point (0.7,0.5,0.4) is on the intersection of these two surfaces. Substituting initial value (0.3,0.5,0.4) into equations (2.28) and (2.29), respectively, it can be obtained the same $c_{1}$ and $c_{2}$. It means that in the phase space, either point (0.7,0.5,0.4) and point (0.3,0.5,0.4) lie on the same closed curve, or two invariant surfaces intersect two closed curves, and they lie on two different closed curves. As shown in figure 1*a*, it is clearly the latter. The evolution trajectory of the points on these two closed curves over time can be seen in figure 1*b*, from which it can be further determined that the two closed curves are closed orbits centred on interior equilibrium points $\left({\tilde{x}}_{1},\frac{1}{2},\frac{1}{2}\right)$ and $\left({\tilde{x}}_{2},\frac{1}{2},\frac{1}{2}\right)$, respectively. That is, in each of the three populations, both strategies fluctuate and coexist.

In fact, the stable interior equilibrium points of the system given in equation (2.23) are not only $\left(\tilde{x},\frac{1}{2},\frac{1}{2}\right)$ with $\tilde{x}\in (0,\frac{1}{2})\cup (\frac{1}{2},1)$, according to table 2, interior points $\left(\frac{1}{2},\frac{1}{2},\tilde{z}\right)$ with $\tilde{z}\in (\frac{1}{2},1)$ exist and also are stable interior equilibrium points. Under certain initial conditions, there are also some closed orbits in phase space centred on interior equilibrium points $\left(\frac{1}{2},\frac{1}{2},\tilde{z}\right)$ with $\tilde{z}\in (\frac{1}{2},1)$, which the interested reader can verify.

## 3. Conclusion

This paper explores a special class of 2×2×2 asymmetric evolutionary games that satisfy fixed conditions; that is, the determinants of three payoff difference matrices are all zero. Furthermore, it is easy to determine two interior equilibrium points, i.e. $\left(\frac{c_{22}}{c_{22}-c_{12}},\frac{a_{22}}{a_{22}-a_{12}},\frac{b_{22}}{b_{22}-b_{12}}\right)$ and $\left(\frac{b_{22}}{b_{22}-b_{21}},\frac{c_{22}}{c_{22}-c_{21}},\frac{a_{22}}{a_{22}-a_{21}}\right)$. Through the Jacobi matrix local stability analysis method, we prove that these two interior equilibrium points are unstable when system parameters satisfy three inequalities in equation (2.5). If equation (2.5) does not hold, that is, if at least one of the three inequalities is an equality, the stability of interior equilibrium points $\left(\frac{c_{22}}{c_{22}-c_{12}},\frac{a_{22}}{a_{22}-a_{12}},\frac{b_{22}}{b_{22}-b_{12}}\right)$ and $\left(\frac{b_{22}}{b_{22}-b_{21}},\frac{c_{22}}{c_{22}-c_{21}},\frac{a_{22}}{a_{22}-a_{21}}\right)$ cannot be judged by the research method in this paper, which can be left as a concern for future work. However, it is interesting that there may be an infinite number of interior equilibrium points in the system at this time (one component of the expression of the two interior equilibrium points above is any number within the interval (0,1)). In this paper, the generalized Hamilton system theory is used to discuss the stability of these interior equilibrium points, and the results are summarized in table 2. The stable interior equilibrium point discussed here is a nonlinear centre, which indicates that if there is a stable interior equilibrium point in the system, then under some initial conditions, the resulting state is that both strategies coexist in each of the three populations and the proportion of strategies fluctuates.

We prove that the stability of interior equilibrium points is based on generalized Hamiltonian system theory, that is, theorem 4 in this paper. From theorem 6, it is evident that the existence conditions of interior equilibrium points can conclusively demonstrate the Hamiltonian structure of the system under consideration, rendering it a bi-Hamiltonian system. Consequently, within the system, there exists both a Hamiltonian function and a Casimir function. Furthermore, the stability of the equilibrium points can actually be judged by the energy-Casimir function method [57], which is an extension of the Lagrange–Dirichlet method. However, this method has an obvious drawback, i.e. the explicit expression of the Casimir function is generally not easy to obtain. Hence, this paper adopts a way to prove the stability of equilibrium points without solving the Casimir function. In the example, we show the Casimir function is solvable. Both the Casimir function and the Hamiltonian function are conserved quantities of the system. In phase space, they characterize two different invariant surfaces, and the closed curves where these two surfaces intersect are centred on stable interior equilibrium points.

Based on the conclusions obtained in this paper, stable interior equilibrium points can be directly judged according to parameter values in three payoff difference matrices. The research in this paper cannot ensure a comprehensive discussion of all interior equilibrium points in the system, therefore, more dynamic properties need to be further explored. In addition, the research here is only limited to a class of 2×2×2 asymmetric game evolution systems that meet special conditions, i.e. the determinants of three payoff difference matrices are all zero. Hence, a more general 2×2×2 asymmetric evolutionary game will be the focus of future work to study.

## Data Availability

This article has no additional data.

## Appendix A

In the 2×2×2 asymmetric games, suppose that there are three populations A, B and C, and each population has two strategies. The strategy set of population A is $H_{A}=\{A_{1},A_{2}\}$, the strategy set of population B is $H_{B}=\{B_{1},B_{2}\}$ and the strategy set of population C is $H_{C}=\{C_{1},C_{2}\}$. The payoff matrix of this game is shown in table 1.

The fitness functions are then

(A 1)
$$\begin{array}{c} \phi_{i}^{1}\left(\vec{y},\vec{z}\right)=\Pi^{1}\left(e_{i},\vec{y},\vec{z}\right)\\ \phi_{i}^{2}\left(\vec{x},\vec{z}\right)=\Pi^{2}\left(\vec{x},e_{i},\vec{z}\right)\\ \phi_{i}^{3}\left(\vec{x},\vec{y}\right)=\Pi^{3}\left(\vec{x},\vec{y},e_{i}\right) \end{array},$$

where $\Pi^{k}(\cdot ,\cdot ,\cdot )$ is a (0,3) payoff tensor, and refer to Song *et al*. [34] for detailed information, $\vec{x}=\langle x,1-x\rangle$ with $\vec{y}$ and $\vec{z}$ defined similarly. In this case, the dynamics are given by the compact form

(A 2)
$${\dot{w}}_{i}^{k}=w_{i}^{k}[\phi_{i}^{k}-{\bar{\phi }}^{k}],$$

with $\vec{w}=\langle x_{1},x_{2},y_{1},y_{2},z_{1},z_{2}\rangle$ and

(A 3)
$${\bar{\phi }}^{k}=\sum_{i=1}^{2}w_{i}^{k}\phi_{i}^{k}(\vec{w}),$$

and $w_{1}^{1}=x,w_{2}^{1}=1-x,w_{1}^{2}=y,w_{2}^{2}=1-y,w_{1}^{3}=z,w_{2}^{3}=1-z.$
